# The Relationship Between Motivation and Academic Performance Among Medical Students in Riyadh

**DOI:** 10.7759/cureus.46815

**Published:** 2023-10-10

**Authors:** Khalid A Bin Abdulrahman, Abdulrahman S Alshehri, Khalid M Alkhalifah, Ahmed Alasiri, Mohammad S Aldayel, Faisal S Alahmari, Abdulrahman M Alothman, Mohammed A Alfadhel

**Affiliations:** 1 Family and Community Medicine, Imam Mohammad Ibn Saud Islamic University (IMSIU), Riyadh, SAU; 2 Medicine, Imam Mohammad Ibn Saud Islamic University (IMSIU), Riyadh, SAU; 3 Family Medicine, Imam Mohammad Ibn Saud Islamic University (IMSIU), Riyadh, SAU; 4 Psychiatry, Imam Mohammad Ibn Saud Islamic University (IMSIU), Riyadh, SAU

**Keywords:** ability-performance motivation, family medicine, analyzing, evaluation of performance, medical school students

## Abstract

Background: Motivation is the process whereby goal‐directed activities are initiated and sustained. Motivation is a crucial factor in academic achievement. The study aims to measure students' demographic factors and external environments' effect on their motivation and determine the impact of students' motivation and self-efficacy on their learning engagement and academic performance.

Methodology: This is a cross-sectional study that involved distributing an online digital questionnaire, which was applied in the capital of Saudi Arabia, "Riyadh." The students’ motivation was assessed using three scales that are designed to measure the students' intrinsic/extrinsic motivation, self-efficacy, and learning engagement.

Results: In this study, we collected 429 responses from our distributed questionnaire among medical students where males represented 60.1% of the sample. Moreover, we classified the satisfaction level into five subcategories: very satisfied, satisfied, neutral, unsatisfied, and very unsatisfied. We found that most of the students (38.7%) were satisfied with their academic performance, while 17.7% were strongly satisfied. The mean enrollment motivation score in this study was 19.83 (SD 2.69), and when determining its subcategories, we found that the mean intrinsic motivation score was 10.33 (out of 12) and the mean extrinsic motivation score was 10.23 (out of 12). Moreover, the mean self-efficacy score was 9.61 and the mean learning engagement score was 8.97 (out of 12). Moreover, we found that a longer duration needed by the students to reach the college from their residence is significantly associated with lower learning engagement reported by the students (8.54 vs. 9.13 in shorter times, P=0.034). Finally, we found that students who entered medical school as their first choice had significantly higher intrinsic motivation, extrinsic motivation, self-efficacy, and learning engagement.

Conclusion: A student's preference for entering medical school will affect their motivation, self-efficacy, and learning engagement. Moreover, intrinsic and extrinsic motivations significantly correlate with self-efficacy and satisfaction with academic performance; however, they have no effect on the grade point average (GPA) of the last semester. The only factor that positively correlates with students' GPA is learning engagement.

## Introduction

"Motivation is the process whereby goal‐directed activities are initiated and sustained"[1]. However, there is no consensual definition of motivation regarding the dozens of theories built around the concept. Among these, the social cognitive approach has gained considerable importance in studying motivation because it is considered a highly integrative and holistic way of understanding the concept of motivation to learn. According to this approach, motivation to learn is determined by both the individual himself and the environment. More precisely, it results from the constant interaction between a student's perceptions of his learning environment, learning behavior, and environmental factors [2]. All human beings share the motivation to secure their basic survival needs, including communication with each other, food, water, sex, and adaptation. To achieve these needs, motivation is a fundamental requirement at the right time. The concept of motivation is a useful summary concept for how the organism's internal physiological states, current environmental conditions, and the organism's history and experiences interact to modulate goal-directed activity [3].

Motivation is a crucial factor in academic achievement [4]. Precisely, the higher the motivation of medical students, the better their quality of learning, their learning strategies, persistence, and academic performance [2]. Motivation is a concept that has attracted researchers for many decades. Medical education has recently become interested in motivation, having always believed that medical students should be motivated because of their involvement in highly specific training, leading to a particular profession. However, medical students who have an absence of motivation are discouraged and have lost interest in their studies, with a feeling of powerlessness or resignation [2].

Academic motivation is one of the concepts studied with respect to student engagement. A previous study conducted by Skinner et al. looked at student participation as a result of their initiatives [5]. In addition, without engagement, there is no effective psychological cycle in learning and development. Moreover, Dörnyei found that students, even those with a high level of self-efficacy, find it difficult to understand the whole unless they are actively involved in learning [6]. Lin discussed the relationship between academic motivation and student engagement and considered academic motivation as a form of discipline that affects a person's behavior positively or negatively [7]. In addition, academic motivation, along with student involvement, influences one's goals, past experiences, cultural background, and the opinions of teachers and peers. Self-efficacy expresses one's belief in overcoming adversity [8]. Bandura et al. defined the word as an individual achieving the desired academic results. If students believe they can complete a task, they are more likely to engage in it. After Bandura et al. introduced the definition, the relationship between self-efficacy and academic success was discovered [9]. According to the results of the study, students with high levels of participation are more self-efficient than students with low levels of participation; It has been observed that these students spend a lot of time learning [10].

The impact and the influence of motivation on students' academic achievements and how motivation plays a vital role in learning have been well researched; many well-conducted studies over the past decades have shown that students' motivation has a high positive correlation with their academic performance. Internationally, a recent cross-sectional study in China in 2020 investigated the relationships between medical students' motivation and self-efficacy, learning engagement, and academic performance. They collected data from 1930 medical students by using an electronic questionnaire and data provided by their institutions; they found that the effectiveness of intrinsic motivation (e.g., if they have a strong interest in medicine) on academic performance is larger than that of extrinsic motivation (e.g., if their family or friends strongly encourage them to choose medicine). The direct effect of self-efficacy on academic performance was not significant. In addition, in this study, gender plays an important role. They found that male students have higher intrinsic motivation but surprisingly lower academic performance in comparison to females [11]. In a subsequent cross-sectional study conducted in 2018, 4,290 medical students from 10 countries in Latin America were among the students. This study investigates if the motivation that pushed Latin American students to choose a medical career is associated with their academic performance during their medical studies [12].

Different types of motivation have been shown to positively impact study technique, academic performance, and adjustment in students in education areas other than medical education [13]. Studying motivation, especially in medical students, is very important because clinical education is not quite the same as general education in different aspects. Some of them require clinical work alongside study. A recent study in the Netherlands created motivational profiles of medical students using high or low intrinsic and controlled motivation. It assessed whether different motivational profiles are associated with various academic performance results. They found high intrinsic motivation with low controlled motivations related to great study hours, deep learning strategy, good academic performance, and low exhaustion from studying. High intrinsic high controlled motivation was also associated with a good learning profile, except that those students with this profile showed high surface strategy. Low intrinsic high controlled and low intrinsic low controlled motivation was related to the least desirable learning practice [14]. Another study conducted in Iran investigated the relationship between academic self-efficacy and academic motivation among Iran's medical science students. Two hundred sixty-four undergraduate students at Qom University of Medical Sciences were selected through a random sampling method. They completed a questionnaire consisting of three sections: demographic characteristics, academic motivation, and academic self-efficacy. They found that achievement scores at the end of each semester and all scores on self-efficacy were altogether associated with academic motivation, while there was no noteworthy relationship between some demographic factors (e.g., age, gender) and academic motivation [15]. That confidence in academic performance outside of the classroom resulted in students' success. Such performance encourages the student to have faith in themselves and their self-efficacy and be more academically motivated. As time goes on, year after year, students lose their motivation.

Our study aims to measure students' demographic factors and external environments' effect on their motivation and determine the impact of students' motivation and self-efficacy on their learning engagement and academic performance. With that being stated, we believe motivation is a critical aspect of elevating academic performance. We aim to explore the relationship between motivation and academic performance in Riyadh medical schools to promote motivation and improve academic performance and outcomes.

## Materials and methods

Study design and setting

This is a cross-sectional study that involved distributing an online digital questionnaire, which was applied in the capital of Saudi Arabia, "Riyadh."

Study subjects

The study population is all current medical students in medical schools in Riyadh, Saudi Arabia. The sample size was estimated via calculation using the sample size formula to assume that the number of medical students in Riyadh is 6,000, 95% confidence level, and 5% margin of error resulting in a sample size of 362. Inclusion criteria encompassed all current medical students in Riyadh, while students outside Riyadh were excluded.

Study tools

In this study, we depended on the questionnaire that was validated and used in a previous study conducted in a different setting [11]. The questionnaire consisted of two main parts: the first part included questions about the demographic factors of the students including gender, level, time needed from student’s residency to reach the university, and method of admission to medical school. The second part was divided into three parts including enrollment motivation, self-efficacy, and learning engagement. The three subscales were designed to measure the students' intrinsic/extrinsic motivation, self-efficacy, and learning engagement, respectively. In particular, the enrolment motivation scale was adapted from the academic motivation scale (AMS) [16]. The AMS scale consisted of 20 items which represent 42.2% of the total variance and discovered three factors: self-discovery, using the knowledge, and discovery. Internal consistency changed between 0.72 and 0.88 in both factors, and the total scale’s Cronbach alpha value was 0.92 [17], while the learning engagement scale was adapted from the Utrecht Work Engagement Scale (UWES) for students [18]. The UWES-9S is a nine-item self-report scale grouped into three subscales with three items each: vigor, dedication, and absorption [19]. All items were scored on a seven-point frequency rating scale ranging from 0 (never) to 6 (always).

Statistical analysis

The collected data was cleaned, entered, and analyzed using SPSS Statistics version 23 (IBM Corp. Released 2015. IBM SPSS Statistics for Windows, Version 23.0. Armonk, NY: IBM Corp.). Frequency and percent were used for the description of categorical variables, while mean, SD, maximum, and minimum were used for the description of ongoing variables. ANOVA test was used to find the correlation between the scores of both tools with the status of vision. All statements were considered significant if the p-value was less than 0.05.

Ethical consideration

The study was conducted after receiving ethical approval from Imam Mohammed Ibn Saud Islamic University, College of Medicine (19-2021). All patients had to provide consent before participating in the questionnaire.

## Results

In this study, we collected 429 responses from our distributed questionnaire among medical students with an 85% response rate, where males represented 60.1% of the sample. Considering the marital status of the students, we found that almost all of the students were single. Moreover, 41.7% of students claimed that getting to their medical school from their residence required them to travel for 15 to 30 minutes each day, while 31.9% needed less than 15 minutes and 26.3% needed more than half an hour. Furthermore, 24.9% of the students were in year 1, while 23.1% were in year 3, and 22.8% were in year 2. Moreover, we found that 93.7 % of the students entered medical school as their first choice and 41.3% indicated that they had a grade point average (GPA) of 4.75-5 in the last semester. Furthermore, we found that 71.8% of the students thought that they had the complete motivation to complete their education, whereas family members were the main persons who gave them the motivation (69%), as shown in Table 1.

**Table 1:** Demographic factors of the students (N=429) GPA: grade point average

		Count	Column N %
Gender	Male	258	60.1%
	Female	171	39.9%
Marital status	Single	422	98.4%
	Married	4	0.9%
	Divorced	3	0.7%
How long does it take from your residence location until you reach the college?	15 minutes or less	137	31.9%
	Between 15 and 30 minutes	179	41.7%
	30 minutes or more	113	26.3%
Grade	Year 1	107	24.9%
	Year 2	98	22.8%
	Year 3	99	23.1%
	Year 4	56	13.1%
	Year 5	69	16.1%
Method of admission	Medicine was my first choice	402	93.7%
	Medicine was NOT my first choice	27	6.3%
GPA of the last semester	<3.5	32	7.5%
	3.5-3.99	65	15.2%
	4-4.49	77	17.9%
	4.5-4.74	78	18.2%
	4.75-5	177	41.3%
Do you think that you have the complete motivation to complete your education?	No	22	5.1%
	To some extent	99	23.1%
	Yes	308	71.8%
Who is the main person who gives you the motivation to complete your education?	Family members	296	69.0%
	Friends and teachers	47	11.0%
	No one (own motivation)	86	20.0%

**Figure 1:**
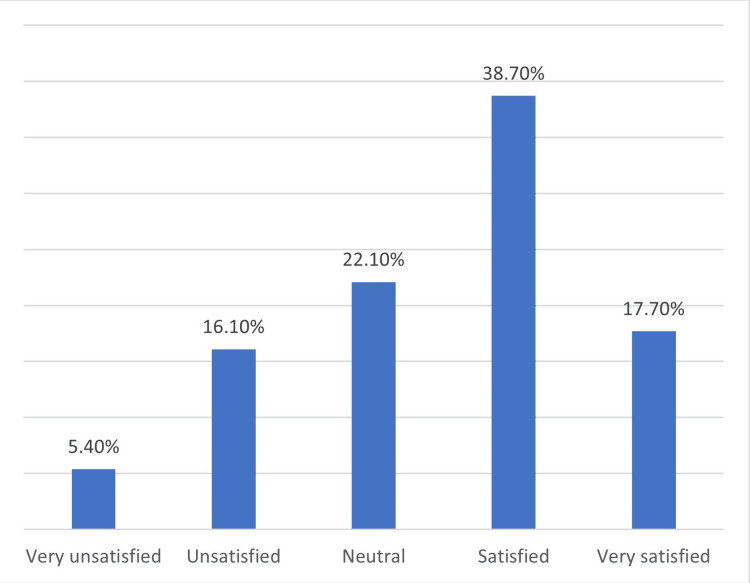
Students' satisfaction with their academic performance

As represented in Figure 1, we found that 38.7% of the students were satisfied with their academic performance, while 17.7% were strongly satisfied; however, 16.1% of the students were unsatisfied with their academic performance, while 5.4% were very unsatisfied.

Moreover, in this study, we used three scales to assess three factors of the students including their enrollment motivations, self-efficacy, and learning involvement. Enrollment motivation scores in this study ranged from 8 to 24 with a mean score of 19.83 (SD: 2.69), and when determining its subcategories, we found that the mean intrinsic motivation score was 10.33 (out of 12), and the mean extrinsic motivation score was 10.23 (out of 12). Moreover, the self-efficacy score ranged from 2 to 12 with a mean score of 9.61, and the mean learning engagement score was 8.97 (out of 12), as shown in Table 2.

**Table 2:** Mean, SD, minimum, and maximum scores of the three scales including enrollment motivation, self-efficacy, and learning engagement

	Mean	SD	Minimum	Maximum
Enrollment motivation	19.83	2.69	8.00	24.00
Intrinsic motivation	10.23	1.47	4.00	12.00
Extrinsic motivation	9.60	1.83	0.00	12.00
Self-efficacy	9.61	1.82	2.00	12.00
Learning engagement	8.97	2.07	1.00	12.00

In this study, we found that entering medical school as the first choice or not significantly affects enrolment motivations, where students who entered medical school as their first choice had significantly higher intrinsic motivation, extrinsic motivation, self-efficacy, learning engagement, students’ motivation, and enrollment motivation. Moreover, we did not find any significant differences among students with different GPAs depending on their motivation, self-efficacy, or learning engagement. However, when dividing the students into two groups with GPAs lower and higher than 4.5, we found a significant difference between the two groups. Intrinsic motivation was significantly higher in students with higher GPAs than other students (10.35 vs. 10.06, P=0.04), as well as considering self-efficacy where students with GPAs higher than 4.5 reported a higher level of self-efficacy (9.79 vs. 9.34, P=0.012) and higher learning engagement in students with GPAs higher than 4.5 (9.15) than those with GPAs lower than 4.5 (8.72) (P=0.035). Extrinsic motivation was higher in students with higher GPAs but with no significant difference (P=0.175). Considering students’ enrollment motivation, we found that students with higher GPAs have a higher level of enrollment motivation and a significantly higher level of students’ motivation than those with lower GPAs (Table 3).

**Table 3:** The relationship between students’ demographic factors and their motivation, self-efficacy, and learning engagement * Significant at p-value less than or equal to 0.05 IM: intrinsic motivation, EM: extrinsic motivation, SE: self-efficacy, LE: learning engagement, GPA: grade point average

		IM	EM	SE	LE	Students’ motivation	Enrollment motivation
		Mean	Mean	Mean	Mean	Mean	Mean
Method of admission	Medicine was my first choice	10.32	9.65	9.63	9.03	29.59	19.97
	Medicine was NOT my first choice	8.96	8.85	9.30	8.19	27.11	17.81
	P-value	0.00*	0.028*	0.048*	0.041*	0.000*	0.001*
GPA of the last semester	<3.5	10.47	9.59	9.50	8.66	29.56	20.06
	3.5-3.99	10.02	9.49	9.35	8.54	28.86	19.51
	4-4.49	9.92	9.36	9.26	8.90	28.55	19.29
	4.5-4.74	10.21	9.78	9.96	9.14	29.95	19.99
	4.75-5	10.42	9.66	9.71	9.15	29.79	20.08
	P-value	0.072	0.649	0.101	0.237	0.190	0.07
GPA (divided into two categories)	<4.5	10.06	9.45	9.34	8.72	28.85	19.51
	>4.5	10.35	9.70	9.79	9.15	29.84	20.05
	P-value	0.04*	0.175	0.012*	0.035*	0.041*	0.008*

In Table 4, we showed the correlation between enrollment motivation and self-efficacy, learning engagement, and GPA. We found that intrinsic and extrinsic motivation significantly correlate with self-efficacy and learning engagement; however, they had no effect on the GPA of the last semester. The only factor that was positively correlated with the GPA of students was learning engagement, where the higher the learning engagement score of the students, the higher their GPA (Table 4).

**Table 4:** The correlation between enrollment motivation and self-efficacy, learning engagement, and GPA and their satisfaction with academic performance IM: intrinsic motivations, EM: extrinsic motivation, SE: self-efficacy, LE: learning engagement, GPA: grade point average

		IM	EM	SE	LE	GPA of the last semester	To what extent are you satisfied with your academic performance?
IM	Pearson correlation	1	0.324	0.382	0.399	0.074	0.228
	Sig. (2-tailed)	-	0.000	0.000	0.000	0.124	0.000
EM	Pearson correlation	0.324	1	0.299	0.252	0.042	0.158
	Sig. (2-tailed)	0.000	-	0.000	0.000	0.390	0.001
SE	Pearson correlation	0.382	0.299	1	0.314	0.085	0.298
	Sig. (2-tailed)	0.000	0.000	-	0.000	0.079	0.000
LE	Pearson correlation	0.399	0.252	0.314	1	0.105	0.325
	Sig. (2-tailed)	0.000	0.000	0.000	-	0.030	0.000
GPA of the last semester	Pearson correlation	0.074	0.042	0.085	0.105	1	0.515
	Sig. (2-tailed)	0.124	0.390	0.079	0.030	-	0.000

## Discussion

In this study, we aimed to measure the impact of students' demographic factors and external environments on their motivation and determine the impact of students' motivation and self-efficacy on their learning engagement and academic performance.

The results of this study showed that neither the gender, grade of the students, nor how far their residency had an impact on their motivation. Moreover, a student's preference for entering medical school will affect their motivation, self-efficacy, and learning engagement. Moreover, intrinsic and extrinsic motivations significantly correlate with self-efficacy and satisfaction with academic performance but have no effect on the GPA of the last semester. The only factor that positively correlated with the students' GPAs was learning engagement. A study by Javadi et al. found that the mean intrinsic and extrinsic motivation score was higher in females than in males and in freshmen than higher-level students [20]. A study by Wu et al. found that male students reported significantly higher intrinsic motivation but surprisingly lower levels of academic performance than female students [11]. In another study conducted by Kusurkar et al., females had higher intrinsic motivation than males in medical education settings [14]. The only factor that affected the students’ motivation, whether intrinsic or extrinsic motivation, was their willingness to enter medical school; students who reported that entering medical school was their first choice had a significantly higher motivation than those who said it wasn't. Considering demographic factors affecting self-efficacy, we found that students' gender, grade, and willingness to enter medical school are all factors that affect their level of self-efficacy; males, older students, and those who indicated that entering medical school was their first choice all reported having higher levels of self-efficacy. Moreover, we found that there was no difference reported between genders in terms of learning engagement. However, we found that the time it takes to get to the college from residency has a significant impact on learning engagement, where students at farther residency would have lower learning engagement than those at closer residency. This result was also reported in previous studies [21,22]. Furthermore, we found that students who enter medical school as their first choice have a higher level of learning engagement, contrary to those who didn’t choose medical school as their first choice. These results indicated that the main factors affecting student’s motivation, self-efficacy, and learning engagement are their choice and will to enter medical school. Therefore, one of the important recommendations in this study is to let students choose their career destination [23]. In Saudi Arabia, as well as many other Arabic countries, entering medical school is a great achievement from the point of view of parents and society [24]. Therefore, this could put pressure on students to enter medical school even though this is not what they want. According to our study, this will affect their motivation, self-efficacy, and learning motivations.

Moreover, the results of this study showed that intrinsic and extrinsic motivation significantly correlate with self-efficacy, learning engagement, and satisfaction with academic performance but have no effect on the GPA of the last semester. The only factor that positively correlates with the GPA of students was learning engagement where the higher the learning engagement score of the students, the higher their GPA. This indicates that the motivations of the students have a significant impact on their learning engagement and academic performance. These results contradict those of Javadi et al. who found no significant correlation between intrinsic and extrinsic motivation and academic performance [20]. Previous studies, including that of Wu et al., found that intrinsic and extrinsic motivation was significantly and positively associated with self-efficacy and learning engagement. However, in this study, extrinsic motivation had no significant association with the students’ academic performance while intrinsic motivations had [11]. Moreover, these results were also found in other studies including that of Fan et al. [25], Walker et al. [26], Bakker [27], and Baker [28]. Moreover, previous studies confirmed a positive relationship between learning engagement and academic performance found in this study [29,30].

Limitations

This study has some limitations. One of these limitations is the dependence on self-reported questionnaires which could lead to some personal bias including the desire of students to appear better. Moreover, we depended on students’ self-report of their GPA of the last semester which could lead to some bias including remember bias and personal bias where students would tend to report higher scores. The study’s sample size and gender distribution might limit the generalizability of the findings. Addressing these limitations through diverse samples and longitudinal studies would enhance the study’s quality and enrich the insights drawn from its results.

## Conclusions

In conclusion, we found that students’ will to enter medical school is the main factor affecting their motivation, self-efficacy, and learning engagement. Moreover, intrinsic and extrinsic motivations significantly correlate with self-efficacy, learning engagement, and satisfaction with academic performance but have no effect on the GPA of the last semester. The only factor that positively correlates with the students' GPA is learning engagement.
